# The Medical Education of Women

**Published:** 1890-10-18

**Authors:** 


### The Medical Education of Women.

That means are to be provided for the medical education of women, upon the same lines and in substantially the same degree as those used for the medical education of men, and that women who have taken this course and have passed the final examinations are to be allowed to register themselves as physicians and to practice with all the rights and privileges of male physicians, is now a fixed fact so far as Great Britain and the United States are concerned.

Even in Japan the law has now been changed so that women may study medicine and be registered as practitioners, thus returning to the customs of that country of eight centuries ago. At the recent International Medical Congress in Berlin, female physicians coming from countries where women are allowed to register and practise were allowed to register and take part in the work of the sections, the committee on organisations taking the ground that while female physicians are not countenanced in Germany, yet that in an International Congress anyone entitled to medical degrees and practice in any country should be allowed a place in the representation from that country.

Women have their own medical schools both in this country and America. They receive medical degrees, and register and practise as physicians; and enough of them have done this, or are in attendance upon their schools, to prove that there are a number of women who like the work, and that thus far a fair proportion have succeeded in making good incomes by the practice of medicine.

Nevertheless, those who advocate the right of women to receive the same education, and to have the same opportunities of professional work that men have, are by no means satisfied with the arrangements which are now offered to women to fit themselves for the practice of medicine, and say that the only way to make these sufficient, and to give young women the same chances as young men now possess for success as physicians, surgeons, or specialists is co-education. They ask that Universities and medical schools shall ignore distinctions of sex in medical education; that male and female students shall work side by side in the laboratory and dissecting-room, and receive their general and clinical instruction together in the same lecture-rooms, amphitheatres, and wards; that they shall pass the same examinations and compete with each other for the same prizes and clinical positions, be members of the same medical societies, and equally eligible for professional chairs and for medical offices under Government. In Great Britain these demands have not yet become pressing, but in the United States they are receiving careful consideration from the authorities having control of some of the older schools and Universities, and this is due, not so much to increase of appreciation of women's rights in the abstract, as to the fact that their decision may have an important influence upon the revenues of the institutions of which they have charge.

During the present year a movement has been set on foot by a number of ladies in Baltimore to raise the sum of £50,000, which is to be offered to the trustees of the Johns Hopkins University, as a contribution to the endowment of its medical department, on the condition that this department shall be open to women on the same terms and conditions as to men, and it is very probable that the money will be obtained. Several years ago a similar offer, although for a much smaller sum, was made to the governors of Harvard University, but was declined. We have reason to believe that within the next five years similar offers will be made to two or three others of the oldest, largest, and best known medical schools in the United States, and it is probable that these offers are made, and that they will also be considered, very largely from the business point of view. Those who are willing to give or raise money for the medical education of women, think it wiser to make, if possible, the necessary arrangements with an already existing first class institution where they can be sure of getting an equivalent if their offer is accepted, rather than to undertake to organise a new school and provide it with the necessary means for laboratory and clinical teaching. On the other hand, the Johns Hopkins trustees, or the trustees of the University of Pennsylvania when such an offer is made to them will ask, will it pay to do this? Can we keep our distinguished professors, have the same number of the better class of male medical students, and maintain the reputation and prosperity of our school if we admit women and arrange for mixed classes throughout? It is true that these will be only some of the questions they will ask, for some of them will decide the question on purely moral grounds, but after all the conclusion of the whole body will probably be based on considerations of business expediency. The objections to mixed classes relate chiefly to the study of anatomy and to clinical teaching.

We do not at present propose to consider these objections but merely to note the fact that they exist, and that they differ greatly in force in different localities and with different men. The question as to the advisability of mixed classes in medical teaching is therefore not an abstract one and cannot be answered alike for all places and all times. We shall watch with interest for the decisions made by the principal American Medical Schools upon this point within the next two or three years, and in the meantime the wisest thing which can be done by the Medical Schools in this country is to keep the sexes apart and wait for the results of the trial of the mixed class system by our cousins across the sea.

